# High-resolution electron time-of-flight spectrometers for angle-resolved measurements at the SQS Instrument at the European XFEL

**DOI:** 10.1107/S1600577522002284

**Published:** 2022-04-01

**Authors:** Alberto De Fanis, Markus Ilchen, Alexander Achner, Thomas M. Baumann, Rebecca Boll, Jens Buck, Cyril Danilevsky, Sergey Esenov, Benjamin Erk, Patrik Grychtol, Gregor Hartmann, Jia Liu, Tommaso Mazza, Jacobo Montaño, Valerija Music, Yevheniy Ovcharenko, Nils Rennhack, Daniel Rivas, Daniel Rolles, Philipp Schmidt, Hamed Sotoudi Namin, Frank Scholz, Jens Viefhaus, Peter Walter, Pawel Ziółkowski, Haiou Zhang, Michael Meyer

**Affiliations:** a European XFEL, Holzkoppel 4, 22869 Schenefeld, Germany; b Institut für Physik und CINSaT, Universität Kassel, Heinrich-Plett-Strasse 40, 34132 Kassel, Germany; c Deutsches Elektronen-Synchrotron (DESY), Notkestrasse 85, 22607 Hamburg, Germany; d J. R. Macdonald Laboratory, Department of Physics, Kansas State University, Manhattan, KS, USA; e SLAC National Accelerator Laboratory, Menlo Park, CA 94025, USA

**Keywords:** time-of-flight spectrometer, electron spectroscopy, FEL, synchrotron

## Abstract

A set of electron time-of-flight spectrometers for high-resolution angle-resolved spectroscopy was developed for the Small Quantum Systems (SQS) instrument at the SASE3 soft X-ray branch of the European XFEL. The resolving power of this spectrometer design is demonstrated to exceed 10 000 (*E*/Δ*E*), using the well known Ne 1*s* ^−1^3*p* resonant Auger spectrum measured at a photon energy of 867.11 eV at a third-generation synchrotron radiation source. At the European XFEL, a width of ∼0.5 eV full width at half-maximum for a kinetic energy of 800 eV was demonstrated. It is expected that this linewidth can be reached over a broad range of kinetic energies. An array of these spectrometers, with different angular orientations, is tailored for the Atomic-like Quantum Systems endstation for high-resolution angle-resolved spectroscopy of gaseous samples.

## Abstract 2

A set of electron time-of-flight spectrometers for high-resolution angle-resolved spectroscopy has been developed for the Small Quantum Systems (SQS) instrument at the SASE3 soft X-ray branch of the European XFEL.

## 1. Introduction

For decades, electron spectroscopy has been a popular and powerful technique to study the physical and chemical aspects of the interaction between radiation and matter (Siegbahn, 1982 ▸; Schmidt, 1987 ▸).

Electron spectra enable deep insight into the electronic structures of the sample under investigation, as well as into transient electronic states and relaxation processes (Siegbahn, 1982 ▸). At the Small Quantum Systems (SQS) instrument at the European XFEL, irradiation of gaseous samples with ultra-short, very intense X-ray pulses opens the door for studying non-linear phenomena, such as multiple ionization and multi-photon processes, as well as time-resolved experiments following dynamical processes on the femtosecond to atto­second timescale (Grychtol *et al.*, 2021 ▸). Owing to spectrometers mounted in out-of-dipole-plane geometry, this also includes the possibility to study non-dipole phenomena.

Electron spectrometers typically consist of a combination of static electromagnetic fields to disperse electrons of different energies onto a detector, which is either time-sensitive (Hemmers *et al.*, 1998 ▸), position-sensitive (Mårtensson *et al.*, 1994 ▸; Eppink & Parker, 1997 ▸; Cubric *et al.*, 2011 ▸) or both (Ovsyannikov *et al.*, 2013 ▸; Liu *et al.*, 2017 ▸).

In general, numerous high-performance spectrometers are in use covering different applications. For example, hemispherical analyzers (Baltzer *et al.*, 1991 ▸; Mårtensson *et al.*, 1994 ▸) offer the highest energy resolution and are common at synchrotron sources. Magnetic bottle electron spectrometers (MBESs; Kruit & Read, 1983 ▸; Eland & Feifel, 2006 ▸) are also used with synchrotrons, lasers and free-electron lasers (FELs) since they can collect nearly 100% of electrons emitted over the 4π solid emission angle. Spectrometers based on velocity map imaging (VMI) (Eppink & Parker, 1997 ▸) are also common. Like MBESs, they can collect all electrons emitted over the whole 4π solid angle, up to a maximum energy of typically few hundred electronvolts. Their reliance on a 2D position-sensitive detector makes it possible for them to provide both energy and angular information. In many spectrometers aimed at high energy resolution, where the electrons are dispersed in position, the detector relies on a commercial CCD or CMOS camera, often limited in repetition rate well below the kilohertz regime, too slow for FELs operating at megahertz repetition rates (Altarelli, 2007 ▸; Raubenheimer, 2014 ▸; Decking *et al.*, 2020 ▸). If electrons of different energies are dispersed in time, rather than position, the acquisition electronics are sufficiently fast to operate the spectrometers in a shot-resolved mode even at megahertz repetition rates. Position-sensitive detectors capable of operation at megahertz rates can be realized with a micro-channel plate (MCP) fitted with delay-line anodes, as routinely used for COLTRIMS (Ullrich *et al.*, 2003 ▸; Kastirke *et al.*, 2020*a* ▸,*b* ▸) and ARTOF (Ovsyannikov *et al.*, 2013 ▸), for example, but they are generally limited in resolution and/or multi-hit capability.

In an electron time-of-flight (eTOF) spectrometer, particles are focused and retarded by a set of electrostatic components before they reach an MCP detector. The resulting signal is treated either in ‘counting’ mode, if particles hitting the detector are sufficiently separated in time that they can be counted individually, or in ‘analog’ mode, with the analog MCP signal read continuously. For the latter, the replenishing rate of electrons in the individual micro-channels can be a critical parameter: the detector must cope with rapid sequences of highly intense shots, which are common at XFELs. Depending on the charge density in the interaction region at high irradiation levels, the so called space charge and the resulting smearing of the electron flight times poses a challenge to a variety of experimental schemes since it can substantially limit the achievable resolution.

Due to the topical orientation of this work, we will elaborate further about the associated spectroscopic challenges at FELs.

FELs have an inherent pulsed time structure with inter-pulse separations ranging from 200 ns to several milliseconds. At the European XFEL, the shortest separation between two consecutive pulses is 220 ns. Every second, 10 trains are delivered, and each train contains up to 2700 pulses with a repetition rate up to 4.5 MHz (Decking *et al.*, 2020 ▸). However, due to the stochastic nature of FELs using the scheme of self-amplified spontaneous emission (SASE) (Madey, 1971 ▸), different pulses can vary in pulse energy, arrival time and spectro-temporal structure (Hartmann *et al.*, 2018 ▸). Therefore, in many spectroscopic experiments at FELs, each spectrum must ideally be associated with the varying properties of the incoming radiation.

In this regard, eTOF spectrometers offer a variety of unique advantages for their use at high-repetition-rate FELs and have been used already at several facilities (Bozek, 2009 ▸; Young *et al.*, 2010 ▸; Fang *et al.*, 2010 ▸; Cryan *et al.*, 2010 ▸; Kanter *et al.*, 2011 ▸; Braune *et al.*, 2018 ▸; Fukuzawa *et al.*, 2018 ▸; Mazza *et al.*, 2020 ▸).

In the present case, angle-resolved electron spectroscopy with multi-hit capability even at high pulse-repetition rates, large accessible energy windows and high energy resolution, is realized by mounting six identical eTOF spectrometers at different angles around the interaction region of the AQS endstation.

## 2. Preliminary considerations and design

**Figure 1:**
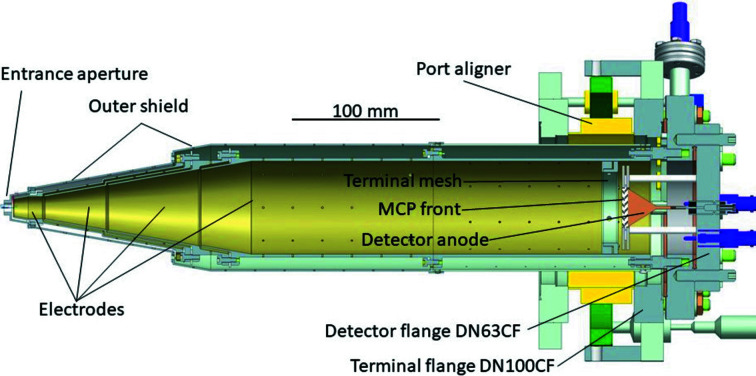
CAD-cutout of the spectrometer. From left to right are the aperture, the electrodes of the conical lens and the drift tube, the mesh, the detector and its conical anode, the flanges with alignment mechanism, and electric feedthroughs.

The concept of the spectrometer presented here shares some of the most common features amongst eTOF spectrometers in general (Hemmers *et al.*, 1998 ▸; Moreschini *et al.*, 2008 ▸). A CAD-cutout of the spectrometer is shown in Fig. 1 ▸. An aperture at the front of the spectrometer selects only electrons emitted over a narrow solid angle. After passing through this aperture, electrons travel through an electrostatic lens before entering a drift tube that ends in a detector assembly. The lens bends the trajectories of all off-axis electrons, increasing the number of electrons reaching the detector rather than landing on some inner surface of the spectrometer. This also reduces background from secondary electrons.

A negative bias on the drift tube retards the electrons, increasing their flight times and thus the time-to-energy dispersion, and therefore improving the energy resolution. At the end of the drift tube, a few millimetres before the detector, is a fine copper mesh (transmission 88%, thickness 12 µm). The mesh ensures that the area inside the drift tube remains field-free when the front of the detector is at a different potential. The bias on the front of the MCP ensures electrons land with sufficient energy to be detected.

This whole structure is enclosed in an electrostatic shield to prevent the field of the spectrometer ‘leaking out’. Each of the five electrodes (the drift tube, the three-element lens and the shield) can be independently biased. Any ripples in focusing and retarding voltages translate into a broadening of the electron spectra, and it can therefore be important for high-resolution measurements to add low-pass filters, not present at the moment, to the power supplies providing the focusing and retarding voltages.

**Figure 2:**
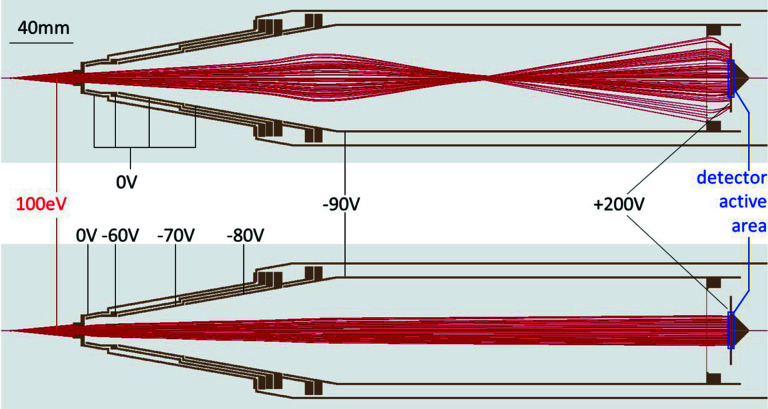
Effect of focusing and retarding voltages on the simulated particle trajectories.

The front part of the spectrometer has a narrow conical profile, with a ±10° angle, so it is possible to mount more than one spectrometer observing the same interaction point. This conical shape differs from many other designs (Hemmers *et al.*, 1998 ▸; Paulus *et al.*, 2006 ▸; Moreschini *et al.*, 2008 ▸; Lebedev *et al.*, 2008 ▸; Jozwiak *et al.*, 2010 ▸), where a sequence of consecutive cylindrical shapes with increasing radius is preferred. The advantage of conical profiles, adopted here from the design of Viefhaus and coworkers (Ilchen *et al.*, 2014 ▸), is that the electron trajectories remain far from the inner surfaces of the spectrometer, offering better control of the trajectories and hence increased transmission. The lens and the front of the spectrometer ideally should start as close as possible to the interaction volume to give better control of the electron trajectories, while at the same time leaving sufficient space for other parts of the experiments, in this specific case, for example, gas delivery and beam monitors. An illustration showing the effect of focusing and retardation voltages on the simulated particle trajectories is shown in Fig. 2 ▸, where electrons with initial energies of 100 eV are retarded to 10 eV in the drift tube. Since electrons that land outside the small active area of the detector are not detected, the benefit of favorable focusing for increasing the count rate is evident from the figure.

The front of the spectrometer is an aperture of 20 mm outer diameter, at a distance from the source of approximately *z* = 40 mm. This aperture has an inner diameter *d* = 6 mm, resulting in a solid acceptance angle of (*d*/*z*)^2^/16 = 0.14% of 4π, or ±4.3°. The outer diameter of the spectrometer enclosure reaches a maximum of 88 mm. The detector is at a distance *L* = 417 mm from the aperture and has an active area with 27 mm diameter.

At its end, the spectrometer is mounted on a DN100CF flange with tip and tilt degrees of freedom for mechanical alignment. The final flange provides ports for feeding the ≤5 kV focusing/retarding voltages into the vacuum environment. The design and materials of the spectrometer allow us to rapidly reach a chamber background pressure of 10^−7^ Pa after a modest bakeout (24 h at 85°C) or 10^−8^ Pa after days.

## 3. Simulations

Charged-particle optics simulations were performed before the spectrometer was designed. They helped to define the overall length *L*, the length of each individual electrode (lens, drift tube) and the opening size of the aperture. The length of the spectrometer plays a crucial role in the trade-off between resolution and transmission. For unretarded electrons of mass *m* and energy *E*, the time-to-energy dispersion is 

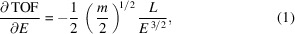

suggesting that longer spectrometers can offer better dispersion and consequently narrower bandwidth. However, shorter spectrometers are more tolerant to residual magnetic fields (see explanation below for more details), and they also have a larger solid angle observed by the detector.

The commercial program SIMION was used for the simulations, combined with a custom script to run nested loops over the focusing and retarding voltages. A number of different spectrometer designs were tested, all with five independent electrodes (three for the lens, one drift tube and one shield) and front ±10° conical profile. The differences between the designs considered are the size of the entrance aperture and the length of each lens segment. For each design, the outcome of the simulations is a large collection of trajectories, from which resolution and transmission are extracted. Electrons with very similar energies *E* and *E* + δ*E* are ‘flown’, *i.e.* simulated, and detected after an average time TOF(*E*) and TOF(*E* + δ*E*). From these quantities, the energy width can be expressed as

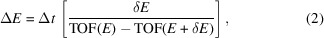

where the overall time width Δ*t* is approximated to the quadrature sum of four contributions from detector and electronics δ*t*_D_, photon pulse duration, as well as jitter of trigger signal, and spread in flight times δ(time-of-flight) for mono-energetic electrons due to different trajectories. With this approach, one obtains many pairs of results (transmission, resolution) for each spectrometer design. From these large datasets, the design that offers the best compromise in terms of transmission and resolution for its dedicated purpose at SQS was selected. The effect of the finite source volume was taken into account, confirming that, for very small sources (<10 µm), the source can be considered point-like.

A narrow energy width Δ*E* is the combined result of narrow time width Δ*t* and large dispersion ∂(time-of-flight)/∂*E*. Large dispersion is given by slow (retarded) electrons in a long spectrometer. But slower electrons in a long spectrometer experience higher off-axis deflection caused by any residual magnetic field. For electrons of energy *E* over a distance *L* inside a magnetic field with component *B*_⊥_ normal to the electron trajectories, this is 

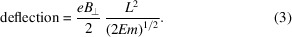

 It is therefore beneficial that the magnetic field throughout the spectrometer is small. For the specific example of Fig. 2 ▸, with 100 eV electrons and 90 V retardation, increasing the component of the magnetic field perpendicular to the spectrometer axis causes the number of detected electrons to decrease linearly to a value of 35 mG, for which no more electrons reach the detector. The typical value of the Earth magnetic field is approximately 0.5 G.

To eliminate the magnetic field around the spectrometer, a set of Helmholtz coils was mounted around it. For our particular spectrometer and electrons with flight times as long as 220 ns, coils with radii >0.5 m are sufficient. Larger coils would ensure transmission for even more retarded electrons, with the benefit of increased dispersion and improved energy resolution.

## 4. Time and energy resolution

The two largest contributions to the time width Δ*t* come from the spread of the trajectories of electrons with identical energies, δ(TOF), and the detector response time δ*t*_D_. Detector and electronic response times which used to be the limiting factors in electron TOF spectroscopy (Hemmers *et al.*, 1998 ▸) are assumed to be state-of-the-art with no contribution from space charge effects.

The spread in flight times δ(TOF) for slower electrons is greater than for faster ones. Due to their larger flight times, however, slower electrons have greater dispersion and ultimately lower Δ*E* than faster ones. When using a detector with very fast response on the order of δ*t*_D_ < 1 ns, the total time width Δ*t* of slow electrons is dominated by the trajectories spread δ(TOF). Hence, for slow electrons, further improvement of the detector response δ*t*_D_ will, after a certain optimum, no longer reduce Δ*E*. On the other hand, the time width Δ*t* of faster electrons is dominated by the detector response δ*t*_D_ (see also further explanations below).

Because of the chromatic nature of all time-of-flight spectrometers, the energy resolution within a given spectrum inevitably changes across the energy range. Simulations were run to determine the magnitude of this change in resolution.

**Figure 3:**
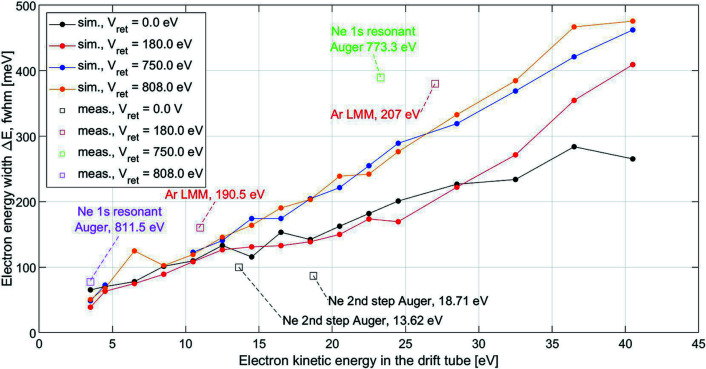
Simulated and measured electron energy widths for different electron energies and different retardation V_ret_. Isolated squares and linked circles are for measurements and simulations, respectively.

The results, in the format of energy width Δ*E* versus energy in the drift tube, are presented in Fig. 3 ▸, for different retardation: 0 V, 180 V, 750 V and 808 V. These retardations were chosen to match the existing measurements (see Section 6). For each retardation, electron energies were considered in the range between 3.5 eV and 43.5 eV above the retardation voltage: 3.5–43.5 eV, 183.5–223.5 eV, 753.5–793.5 eV and 811.5–851.5 eV. For example: the simulated resolution for electrons of initial energy 190.5 eV with a retardation of 180 V is approximately 110 meV, against a measurement of 160 meV. We attribute the remaining disagreement to the limitation in exactly reproducing the spectrometer in the simulation package. The contributions to Δ*E* originating from the finite response time of the detector (δ*t*_D_) and electronics are included in the simulations.

## 5. Detector and data acquisition

The detector, including its electric feed-throughs, is mounted on a DN63CF flange. It can be replaced even when the spectrometer is mounted on the vacuum chamber, without disturbing the mechanical alignment of the spectrometer. The detector is commercially available and based on a pair of micro-channel plates whose active area has diameter of 27 mm. In order to guarantee detection of slow electrons, the front surface of the MCP is typically biased at +200 V. The electron cloud exiting the MCP is collected by a conical copper anode biased at positive high voltage. The analog signal is decoupled from the high positive DC voltage of the anode via an in-vacuum capacitor. Having the capacitor very near the anode facilitates optimum impedance, producing narrower detector signals and thus giving the possibility of higher resolution. The detector response was confirmed to reach the design specifications of 450 ps full width at half-maximum (FWHM). To date, this is exceptionally fast, and it helps achieve high energy resolution, especially for the faster electrons in the spectrum (see previous paragraph), as well as for operating the MCPs in analog mode when multiple electrons are hitting the detector at almost identical times. Depending on the operation mode, the signal can be either processed by counting electronics for histogramming or fed into a fast digitizer. When using counting electronics, it is typically fed to a fast broadband amplifier, shaped and thresholded by a constant fraction discriminator, and then, together with the photon trigger, fed into a time-to-digital converter (TDC). When using a digitizer, the signal can either be used as an analog trace or, if single hits are separated in time, they can be counted individually.

As discussed in previous work focusing on the application at storage-ring-based light sources (Hemmers *et al.*, 1998 ▸), the detector response time, combined with the common pulse duration of ∼100 ps, the jitter of the X-ray trigger on the order of ∼100 ps and the TDC resolution contribute to the measured time width Δ*t* of all electrons, and are particularly significant for fast electrons where δ(TOF) is small.

For high count rates like at FELs, digitizers are normally used. The signal can be preamplified, and is then fed into the digitizer where the whole trace of current over time is recorded. Digitizers currently used at the European XFEL have sampling rates and bandwidths up to 10 GS s^−1^ and 3 GHz, respectively. Using a detector with a response time of 450 ps, the speed of these digitizers ensures they are not further contributing to a loss of energy resolution.

## 6. Commissioning

A prototype of the spectrometers was commissioned at the soft X-ray P04 beamline (Viefhaus *et al.*, 2013 ▸) at the PETRA III storage ring at DESY in Hamburg, Germany, which provides high-flux radiation with a narrow bandwidth focused to a spot of approximately 10 µm × 10 µm. During commissioning, the storage ring operated with pulse-to-pulse separation of approximately 192 ns, similar to the shortest pulse separation at the European XFEL of 220 ns.

The middle point of the spectrometer was in the center of a set of Helmholtz coils with 100 cm diameter. For operation at European XFEL, larger coils have been installed. The gas sample entered the chamber freely via a capillary with a 100 µm inner diameter.

**Figure 4:**
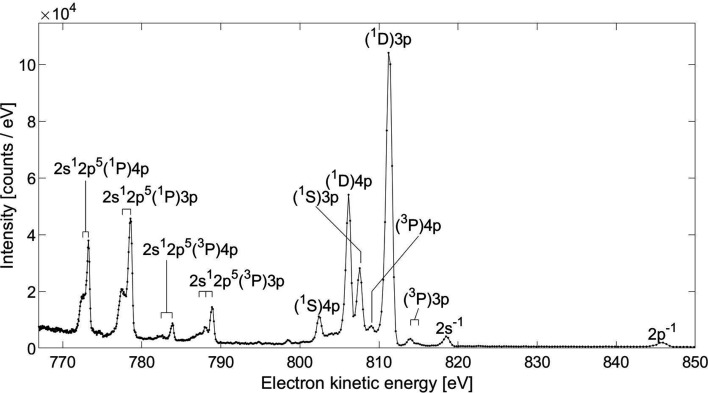
Resonant Auger spectrum of Ne following the 1s^−1^3*p* excitation at 867.17 eV, with 750 V retardation, measured at the storage ring.

Having access to a photon source that can provide sufficient flux in a bandwidth Δ(*h*ν) narrower than the natural lifetime width Γ, resonant Auger spectroscopy is a very convenient way to assess the resolution of an electron spectrometer: the signal yields benefits from the resonant enhancement, and the natural width Γ of the inner-shell vacancy does not contribute to the overall width, unlike in the case of normal Auger or inner-shell photoelectrons (Piancastelli, 2000 ▸). Fig. 4 ▸ shows the 760–850 eV electron kinetic energy range of the Ne 1*s* ^−1^3*p* resonant Auger spectrum measured at a photon energy *h*ν = 867.11 eV with 750 V retardation on the drift tube. The most likely decay of the Ne 1*s* ^−1^3*p* core-excited state is by resonant Auger emission, resulting in an Ne^+^ state with two vacancies in the valence or inner-valence shell, and one Rydberg electron, with the configurations 2*p* ^4^(^3^ *P*, ^1^ *D* or ^1^ *S*)*np*, with *n* = 3 or 4 (Kivimäki *et al.*, 2001 ▸). These Auger lines are visible in Fig. 4 ▸ in the energy range between 800 eV and 816 eV. Other visible Auger lines are those leading to the singly charged final state 2*s* ^1^2*p* ^5^(^1,3^ *P*)*np*, *n* = 3 or 4, in the energy range between 770 eV and 790 eV. In addition, the valence and inner-valence photolines 2*p* ^−1^ and 2*s* ^−1^ are present near 818 eV and 845 eV, respectively. This spectrum is similar in energy range, resolution and intensity distribution to the one in Fig. 7 of Hemmers *et al.* (1998 ▸), which was measured at the same retardation, with a spectrometer of similar length and solid aperture angle.

**Figure 5:**
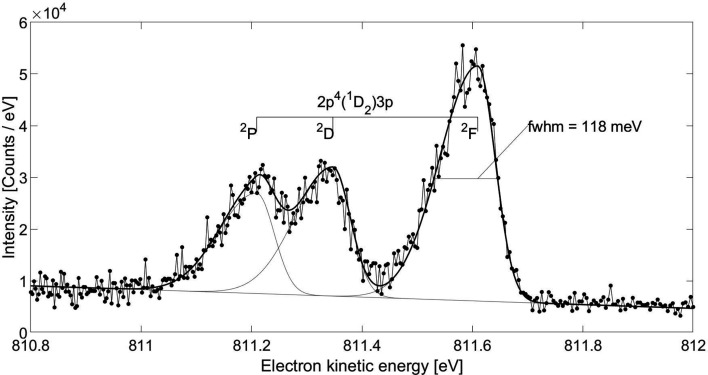
Resonant Auger spectrum of Ne following the 1s^−1^3*p* excitation at 867.17 eV, with 808 V retardation, measured at the storage ring.

The fitted energy width FWHMs in the spectrum in Fig. 4 ▸ are 1.3 eV for the 2*p* ^−1^ photoline near 845 eV [here we ignore the fact that this peak is the combination of its two unresolved components with 96.7 meV separation (Persson, 1971 ▸)], 0.87 eV for the 2*s* ^−1^ photoline near 818 eV and <0.39 eV for the 2*s* ^1^2*p* ^5^(^1^ *P*)4*p* line near 773.4 eV. The resolution can be improved further by increasing the retardation, thereby increasing the dispersion in equation (1). The resulting spectrum, with retardation increased to 808 V, is shown in Fig. 5 ▸, which contains the data and the overall fitting curve as well as individual components. Note that for this higher retardation the flight time of the slowest electrons, about 300 ns, is larger than the inter-pulse separation of 192 ns, causing the spectra originating from consecutive pulses to partly overlap. Each feature of the spectrum in Fig. 5 ▸ is least-square fitted with one asymmetric Gaussian curve sitting on a slanted background. The asymmetric peak is a consequence of the different nature of broadening contributions: within a single energy line, the distribution of time-of-flight for electrons arriving later is broadened by the finite acceptance angle, whereas this broadening is absent for the electrons traveling on axis. The extracted energy width for the lines near 811 eV in the spectrum in Fig. 5 ▸ is approximately 118 meV. After subtracting contributions from the Doppler broadening [= 0.722(*ET*/*M*)^1/2^ = 79 meV with *E* = 811 eV, *M* = 20 a.m.u. and *T* = 293 K] and 39 meV for the photon bandwidth Δ(*h*ν), we obtain a spectrometer bandwidth Δ*E* of only 78 meV, equivalent to the resolving power *E*/Δ*E* > 10 000. Further improvement of the resolution of the spectrometer, for example, by decelerating further in the drift tube, the overall resolution would have a negligible improvement since it is already limited by Doppler broadening. We are not aware of any eTOF spectra at this electron kinetic energy with comparable resolution.

The extracted contributions of the spectrometer to the energy widths, Δ*E*, versus the retarded energy in the drift tube, are compared with the corresponding simulations in Fig. 3 ▸. The text in the figure indicates the electronic transition and the electron energy. The largest discrepancy between simulations and measurements is less than a factor of 2, and in most cases less than 1.5. Given the complexity of the problem, this level of agreement is fair, supporting that the simulations can be useful also at different initial energies. For all energies considered, electrons retarded to the energy of 3.5 eV in the drift tube will have a linewidth Δ*E* < 100 meV. For the largest energy in the drift tube, here 43.5 eV, Δ*E* is still <0.5 eV.

**Figure 6:**
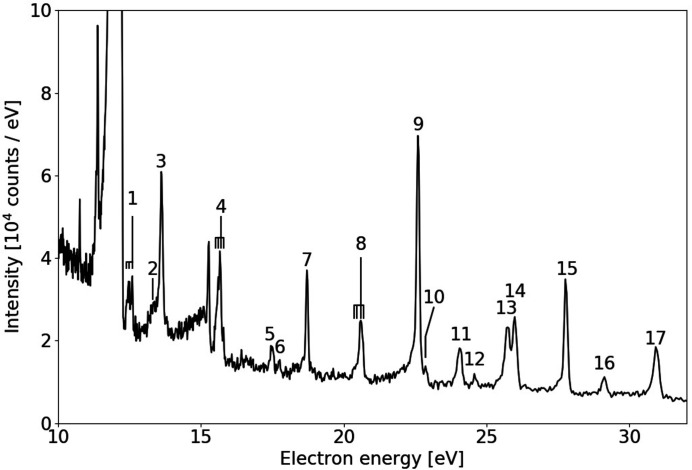
Low-energy part of the resonant Auger spectrum of Ne at a photon energy of 867.17 eV (resonant with the 1*s* ^−1^3*p* excitation), corresponding to electrons from the second step of the Auger cascade. No retardation is applied.

The performance of the spectrometer was also characterized for slow electrons by measuring the spectrum of the Ne 1*s* ^−1^3*p* second step Auger cascade. One such measurement is shown in Fig. 6 ▸ in the electron kinetic energy range between 10 eV and 32 eV.

Most peaks here can be assigned to transitions of the type 2*s* ^1^2*p* ^5^ *np* → 2*p* ^4^ or 2*s* ^0^2*p* ^6^ *np* → 2*p* ^4^, with *n* = 3 or 4 (Yoshida *et al.*, 2005 ▸; Kitajima *et al.*, 2006 ▸; De Fanis *et al.*, 2005 ▸). The assignments are given in Table 1 ▸. Most structures are the combination of unresolved features, with separation as small as a few millielectronvolts (De Fanis *et al.*, 2005 ▸), so their width only indicates an upper limit to the instrumental bandwidth. The best candidate to assess the resolution of the spectrometer in this energy range, in the absence of retardation, is the 2*s* ^0^2*p* ^6^(^1^ *S*)4*p* → 2*s* ^1^2*p* ^5^(^1^ *P*_1_) line at 18.71 eV because it is isolated (Yoshida *et al.*, 2005 ▸): its energy width is 87 meV, even better than the simulations in Fig. 3 ▸. The resolution here is limited by the combination of spread in flight time and dispersion. The former can be reduced by narrowing the entrance aperture and the latter by increasing the flight time by either a longer drift tube or retardation. The structure in Fig. 6 ▸ at an energy between 10.7 eV and 12.3 eV corresponds to fast electrons, photoelectrons and Auger, produced by either a subsequent photon pulse (see also earlier remark on inter-pulse separation), or by higher-order contamination of the beamline. The sharp peak near 15.3 eV is the ‘prompt’ caused by fluorescent photons.

## 7. Operation at the European XFEL SQS instrument

A set of six identical spectrometers in different angular orientation is currently operational at the AQS endstation of the SQS instrument at the European XFEL (Mazza *et al.*, 2020 ▸). Three spectrometers are in the so-called dipole plane, the vertical plane orthogonal to the photon beam. One of these spectrometers is mounted vertically, one horizontally and one at the so-called ‘magic angle’, *i.e.* at 54.7° from the horizontal. The other three spectrometers are upstream of the interaction point, one 45° below the photon beam and the other two either side of it. This configuration allows us to measure asymmetries in the angular distribution patterns, for example caused by non-dipole effects or photoelectron circular dichroism (Hemmers *et al.*, 2004 ▸; Ilchen *et al.*, 2018 ▸; Ritchie, 1976 ▸).

**Figure 7:**
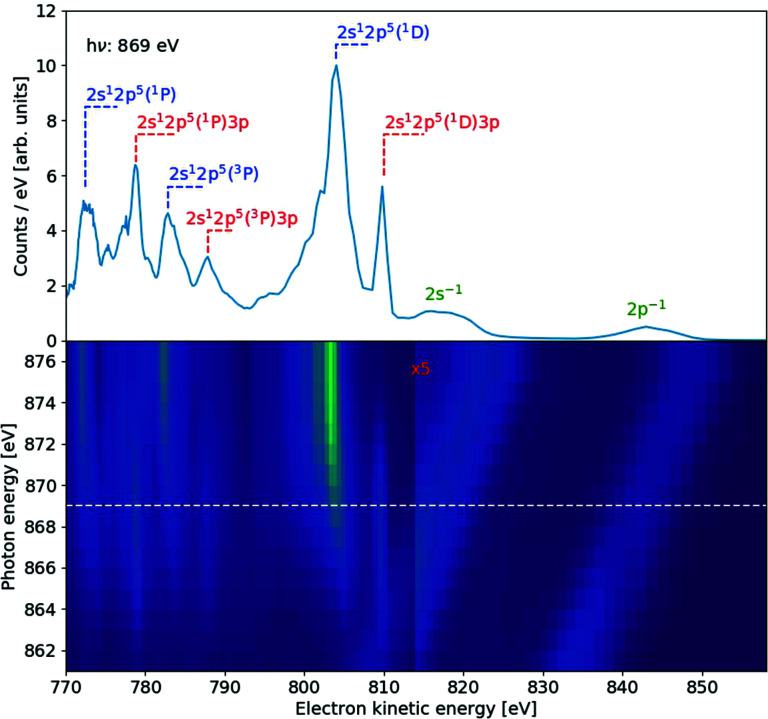
Electron spectra of Ne measured at the SQS instrument in the region of the 1*s* resonances and threshold. The spectrometer is horizontal, perpendicular to the photon beam. Bottom panel: spectra at several photon energies near the 1*s* threshold. The portion at electron energies >813 eV is scaled ×5 for visibility purposes. Upper panel: spectrum at a photon energy of 869 eV, corresponding to the dashed line in the bottom panel. Assignments for some of the peaks are in the upper panel: blue are normal Auger, red are resonant Auger and green are photolines.

An example of measurements from one of these spectrometers, the one horizontal and orthogonal to the FEL beam, with a retardation of 760 V, is presented in Fig. 7 ▸. The region around the resonance processes described above for Fig. 4 ▸ is sampled in photon energy steps of 1 eV. The bottom panel shows a map of electron spectra at kinetic energies in the region 770–857 eV; the upper panel displays the spectrum at a photon energy of 869 eV, corresponding to the dashed line in the lower panel. The beam used here is the ‘pink’ SASE beam from the undulator, non-monochromated, with an ∼1% photon energy bandwidth responsible for broadening the photolines. The two valence photolines 2*s* ^−1^ and 2*p* ^−1^ are assigned to the broad diagonal contributions on the right part of the lower panel. At the 1*s* ^−1^3*p* resonant photon energy of 867 eV, the peaks at electron energies of 778 eV, 804 eV and 811 eV correspond to Auger to the 2*s* ^1^2*p* ^5^(^1^ *P*)3*p*, 2*s* ^1^2*p* ^5^(^3^ *P*)3*p* and 2*p* ^4^(^1^ *D*)3*p* final states, respectively. At the higher photon energies, the normal Auger peaks 2*s* ^1^2*p* ^5^(^1^ *P*), 2*s* ^1^2*p* ^5^(^3^ *P*) and 2*p* ^4^(^1^ *D*) are clearly identified.

**Figure 8:**
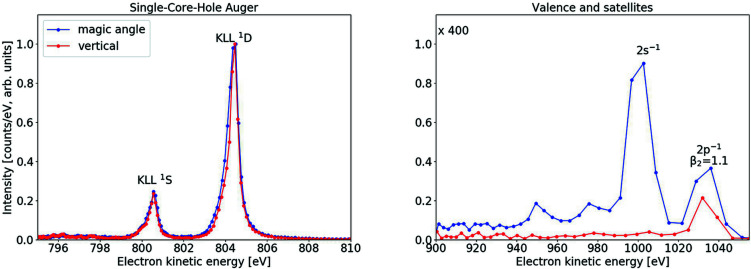
Electron spectra of Ne measured at the SQS instrument with a photon energy of 1050 eV and a retardation of 790 V. The spectrometers are in the dipole plane, vertical (red) and at the magic angle (blue). The spectra on the right are scaled up ×400 for visibility purposes.

Because of the significant photon bandwidth, these features are also visible at photon energies below the threshold. In the threshold region presented, they show broadening and distortion caused by post-collision interaction. The narrowest feature in these spectra is at 778 eV, with a width of ∼0.5 eV. One more example of electron spectra taken at SQS can be found in Fig. 8 ▸, with a photon energy of 1050 eV, retardation of 790 V and attenuated FEL beam to limit space-charge broadening, employing two spectrometers in the dipole plane: the vertical one and the ‘magic angle’ one. The spectra at the two angles are scaled with respect to each other such that the Auger peaks are isotropic. The peaks in the left quadrant are assigned to the Ne *KLL* ^1^ *S* and ^1^ *D* Auger. The *KLL* peaks have an energy width of 0.55 eV, which also includes the natural lifetime width of 0.24 eV. This resolution can potentially be improved further, for example with higher retardation, finer tuning and lower sample density. The peaks in the right panel are the valence photolines and their satellites. The 2*s* ^−1^ photoline is almost completely absent in the 90° spectrum, consistent with the angular distribution for photolines from an *s* orbital, and the intensity ratio for the 2*p* ^−1^ photoline is consistent with a known β_2_ parameter of 1 (Wuilleumier & Krause, 1974 ▸).

One of the six identical time-of-flight spectrometers in the AQS chamber is installed coaxially with a VMI spectrometer around the same interaction point (Mazza *et al.*, 2013 ▸). By biasing these VMI electrodes, this time-of-flight spectrometer is routinely used during user operation for taking high-resolution ion time-of-flight spectra (*e.g.* LaForge *et al.*, 2021 ▸).

## 8. Summary and conclusions

In order to enable high-resolution angle-resolved electron spectroscopy on a pulse-resolved basis at a megahertz repetition rate, new time-of-flight electron spectrometers were simulated, designed, constructed and commissioned. The spectrometer design was tested and confirmed to operate over an electron kinetic energy range up to 845 eV and almost as low as 10 eV. At the electron kinetic energy of 811 eV, a Doppler-limited energy width Δ*E* = 118 meV was measured with a spectrometer contribution of only 78 meV. The outer physical envelope of the spectrometer was chosen such that it is possible to mount several of them looking at the same point in space in order to allow for angle-resolved measurements. The design of the spectrometer was tailored for the SQS instrument at the SASE3 branch of the European XFEL, where it is routinely used, but easily movable and adaptable to other experimental arrangements.

## Figures and Tables

**Table 1:** Assignments of the peaks in Fig. 6 ▸

1	2*s* ^1^2*p* ^5^(^3^ *P*)3*p* → 2*p* ^4^ ^1^ *D*_2_	10	2*s* ^1^2*p* ^5^(^1^ *P*)3*p* ^2^ *P* → 2*p* ^4^ ^1^ *D*_2_
2	2*s* ^1^2*p* ^5^(^3^ *P*)3*p* ^2^ *S* → 2*s* ^2^2*p* ^4^ ^1^ *D*_2_	11	2*s* ^0^2*p* ^6^(^1^ *S*)4*p* → 2*s* ^1^2*p* ^5^ ^3^ *P*
3	2*s* ^0^2*p* ^6^(^1^ *S*)3*p* → 2*s* ^1^2*p* ^5^ ^1^ *P*_1_	12	2*s* ^0^2*p* ^6^(^1^ *S*)3*p* → 2*s* ^1^2*p* ^5^ ^3^ *P*
4	2*s* ^1^2*p* ^5^(^3^ *P*)3*p* → 2*p* ^4^ ^3^ *P*	13	2*s* ^1^2*p* ^5^(^1^ *P*)3*p* ^2^ *D* → 2*p* ^4^ ^3^ *P*
5	2*s* ^1^2*p* ^5^(^3^ *P*)4*p* → 2*p* ^4^ ^1^ *D*_2_	14	2*s* ^1^2*p* ^5^(^1^ *P*)3*p* ^2^ *P* → 2*p* ^4^ ^3^ *P*
6	2*s* ^1^2*p* ^5^(^3^ *P*)4*p* ^2^ *S* → 2*p* ^4^ ^1^ *D*_2_	15	2*s* ^1^2*p* ^5^(^1^ *P*)4*p* → 2*p* ^4^ ^1^ *D*_2_
7	2*s* ^0^2*p* ^6^(^1^ *S*)4*p* → 2*s* ^1^2*p* ^5^ ^1^ *P*_1_	16	2*s* ^0^2*p* ^6^(^1^ *S*)4*p* → 2*s* ^1^2*p* ^5^ ^3^ *P*
8	2*s* ^1^2*p* ^5^(^3^ *P*)4*p* → 2*p* ^4^ ^3^ *P*	17	2*s* ^1^2*p* ^5^(^1^ *P*)4*p* → 2*p* ^4^ ^3^ *P*
9	2*s* ^1^2*p* ^5^(^1^ *P*)3*p* ^2^ *D* → 2*p* ^4^ ^1^ *D*_2_		
